# Recovery of Gelatin from Bovine Skin with the Aid of Pepsin and Its Effects on the Characteristics of the Extracted Gelatin

**DOI:** 10.3390/polym13101554

**Published:** 2021-05-12

**Authors:** Tanbir Ahmad, Amin Ismail, Siti Aqlima Ahmad, Khalilah Abdul Khalil, Elmutaz Atta Awad, Muhammad Tayyab Akhtar, Awis Qurni Sazili

**Affiliations:** 1 Department of Animal Science, Faculty of Agriculture, Universiti Putra Malaysia, UPM, Serdang 43400, Selangor, Malaysia; tanbirvet05@rediffmail.com; 2 ICAR-Indian Veterinary Research Institute (IVRI), Izatnagar, Bareilly 243122, Uttar Pradesh, India; 3 Faculty of Medicine and Health Sciences, Universiti Putra Malaysia, UPM, Serdang 43400, Selangor, Malaysia; aminis@upm.edu.my; 4 Halal Products Research Institute, Putra Infoport, Universiti Putra Malaysia, UPM, Serdang 43400, Selangor, Malaysia; 5 Department of Biochemistry, Faculty of Biotechnology and Molecular Science, Universiti Putra Malaysia, UPM, Serdang 43400, Selangor, Malaysia; aqlima@upm.edu.my; 6 Department of Biology, Faculty of Applied Sciences, Universiti Teknologi MARA, Shah Alam 40450, Selangor, Malaysia; khalilahabdkhalil@gmail.com; 7 Laboratory of Sustainable Animal Production and Biodiversity, Institute of Tropical Agriculture and Food Security, Universiti Putra Malaysia, UPM, Serdang 43400, Selangor, Malaysia; motazata83@gmail.com; 8 Faculty of Veterinary Medicine, Universiti Malaysia Kelantan, Pengkalan Chepa 16100, Kelantan, Malaysia; 9 Institute of Industrial Biotechnology, Government College University, Lahore 54000, Pakistan; tayyabakhtar@hotmail.com; 10 Natural Product Laboratory, Institute of Bioscience, Universiti Putra Malaysia, UPM, Serdang 43400, Selangor, Malaysia

**Keywords:** gelatin, bovine skin, pepsin, free amino acid, FTIR, NMR

## Abstract

Pepsin enzyme was used to pretreat the bovine skin at the rate of 5, 15, and 25 units of enzyme/g of skin to recover gelatin, and the recovered gelatins were referred to as Pe5, Pe15, and Pe25, respectively. The gelatin yield increased significantly (*p* < 0.05) from 18.17% for Pe5 to 24.67% for Pe25 as the level of pepsin increased, but the corresponding gel strength and viscosity decreased significantly (*p* < 0.05) from 215.49 to 56.06 g and 9.17 to 8.17 mPa·s for Pe5 and Pe25, respectively. β- and α1- and α2-chains were degraded entirely in all the gelatins samples as observed in protein pattern elaborated by gel electrophoresis. ^1^H nuclear magnetic resonance (^1^H NMR) analysis indicated the coiled structure of gelatin protein chains. The lowest amide III amplitude of Pe25 as found by Fourier transform infrared (FTIR) spectroscopy indicated that α-helix structure of protein chains were lost to more irregular coiled structure. Thus, it could be summarized that pepsin might be used at the lower level (5 units/g of wet skin) to extract gelatin from bovine skin with good functional properties and at higher level (15/25 units/g of wet skin) to obtain gelatin of industrial grade with high yield.

## 1. Introduction

Gelatin is animal source biopolymer [1] protein extracted from collagen by partially hydrolyzing it. Gelatin is primarily used in the food, cosmetic, and pharmaceutical industries. Being extracted from collagen, gelatin closely resembles collagen in amino acid composition, which is generally a repeating sequence of Gly-X-Y where X and Y are normally proline and hydroxyproline. Collagen is made up of tightly wound triple α helix protein polypeptide chains with molecular weight around 330 kDa, whereas gelatin is a mixture of denatured shorter polypeptide chains exhibiting approximate molecular weight of more than 30 kDa [2]. Each chain of the triple helical structure of collagen is twisted in a left-handed manner, and the three chains intertwine to form a right-handed structure referred to as tropocollagen, having 300 nm length and 1.5 nm width [1]. Generally, various intra- and intermolecular bonds present in collagen make it resistant to acid and heat for conversion into gelatin [3], leading to low gelatin extraction yield [4]. Proteolytic enzymes could aid in the better recovery of gelatin from collagen [4,5,6]. Research has reported gelatin with higher yield with desired rheological and functional properties could be obtained by utilizing proteolytic enzymes.

The gelatin yield increased significantly with the pretreatment of thornback ray (*Raja clavata*) skin by pepsin in glycine-HCl buffer [6]. This was due to the destruction of site of intermolecular bonding of collagen by pepsin [6], and thus effective solubilization of collagen in the skin matrix and easing the extraction of gelatin [4]. Porcine pepsin added at the level of 15 units/g (for 48 h at 4 °C) of alkaline-treated bigeye snapper (*Priacanthus tayenus*) skin resulted in better gelatin extraction yield [4]. The inter- and intramolecular covalent bonds of collagen with lysine and hydroxylysine residues and their aldehyde derivatives are generally found in the telopeptide ends of the triple helix chains, and these ends are specifically hydrolyzed by pepsin, thereby contributing to increased gelatin yield [4]. Gelatin extraction using different concentration of pepsin (5, 10, and 15 U of pepsin/g of alkali-treated skin) led to 6.59, 7.69, and 9.22% gelatin recovery, respectively, from cuttlefish (*Sepia officinalis*) skin, whereas the yield of gelatin was only 2.68% in the absence of pepsin [5]. Thus, the role of pepsin in obtaining higher yield of gelatin was established. Although the percentage of bovine skin collagen converted to gelatin was observed to be 32% using pepsin, the gelatin recovery from skin with and without addition of enzyme was not reported by Chomarat et al. [7]. Moreover, only few analyses such as gel strength, viscosity, turbidity, and SDS-PAGE of the obtained gelatin were carried out by the researchers. Considering the above facts, the gelatin was extracted from bovine skin using different levels of pepsin, and obtained gelatin was characterized by analyzing its various physicochemical and functional properties and molecular structure.

## 2. Materials and Methods

### 2.1. Chemicals

Pepsin enzyme (product code: PB0689) was purchased from NBS Biologicals, Bio Basic Inc., Cambridgeshire, UK. Merck, Darmstadt, Germany supplied the chemicals acrylamide, sodium dodecyl sulphate (SDS), Coomassie brilliant blue R-250, *N*,*N*,*N*′,*N*′-tetramethyl ethylene diamine (TEMED), and 2-mercaptoethanol, as well as chemicals used in NMR measurements such as deuterated water (D_2_O, 99.9%) and dimethyl silapentane sulfonic acid (DSS). Hydroxyproline standard was procured from Agilent Technologies (Santa Clara, CA, USA). Amino acid standards, chromatographic column, mobile phase, and reagents were supplied by Waters Corporation, Milford, MA, USA. In other cases, analytical grade chemicals and reagents were used.

### 2.2. Preparation of Skin

Bovine skins from female Brahma cross (3 to 4 years old) were obtained from a local slaughterhouse located at Shah Alam, Selangor, Malaysia, and carried under ice and kept at −20 °C. The skin was cleaned thoroughly of dirt and blood and stored at −20 °C. The skin was thawed at 4 °C overnight before being used for the experiment.

### 2.3. Gelatin Recovery from Bovine Skin Using Pepsin Pretreatment

#### 2.3.1. Removal of Non-Collagenous Proteins

The non-collagenous proteins were removed by immersing the skin under continuous stirring in 0.1 mol/L NaOH (*w*/*v*) solution for 6 h at room temperature at the skin to a solution ratio of 1:5 (*m*/*v*). The solution was replaced at every 2 h interval. Thereafter, skin was washed thoroughly several times so as to ensure that no traces of alkali remained on the skin.

#### 2.3.2. Gelatin Extraction with the Aid of Pepsin

The skin was soaked in the solution of 1% HCl at room temperature at the skin to solution ratio of 1:10 (*m*/*v*) for 20 h with discontinuous stirring. After soaking, skin was washed extensively with distilled water so that acid was removed completely. Then, the skin was pretreated with pepsin enzyme at the level of 5, 15, and 25 units per gram of wet skin for 48 h at the optimum temperature (37 °C) and pH (3.5) of the enzyme, as specified by the supplier.

Optimum pH solution was prepared, and the swollen skin sample was incubated in it at the ratio of 1: 3 (*m*/*v*) and the required amount of pepsin was added. The mixture was kept in the water bath at the 37 °C and stirred. Upon completion of this step, the mixture was kept in water bath maintained at 90 °C for 15 min to terminate the enzyme activity. Extraction of gelatin was performed in water bath with continuous stirring for 6 h at 60 °C. Thereafter, the obtained solution was filtered with the help of cheese cloth (gauze-like cotton cloth mainly used to drain water during cheese making) and the filtrate was centrifuged for 20 min at 12,800× *g* using Beckman Coulter Avanti J-26 XPI centrifugation instrument. Resultant supernatant was dried using Labconco (FreeZone^18^, Kansas, MO, USA) freeze drier. Finally, the dried gelatin sample obtained was stored at 4 °C for future analysis. The control gelatin sample, results of which are already published [8], was extracted in the same way as that of treatment gelatins, except that the pretreated skin samples were not incubated with the enzyme pepsin. Extraction was performed in triplicate to arrive at the mean.

### 2.4. Analyses of Gelatin

#### 2.4.1. Gelatin Yield

Gelatin yield was determined on the wet basis of skin following the previous methods of researchers [9,10].

(1) $$Gelatin\;yield\;\left(\%\right)=\frac{Freeze\;dried\;gelatin\;weight\;\left(g\right)}{Wet\;skin\;weight\;\left(g\right)}\times 100$$

#### 2.4.2. pH

pH of the gelatins was measured following the method of Eastoe and Leach [11]. We prepared 1% (*m*/*v*) gelatin solution by mixing 0.2 g of sample with 20 mL of distilled water (60 °C). The solution was cooled to room temperature before taking the pH measurement using Mettler Toledo pH meter (AG 8603, Greifensee, Switzerland).

#### 2.4.3. Color

ColorFlexHunterLab purchased from Hunter Associates Laboratory Inc. (Reston, VA, USA) was employed to estimate L* (lightness), a* (redness/greenness), and b* (yellowness/blueness) values of the gelatins. Sample cup made up of glass (64 mm diameter) was filled with sample, and the three-color coordinates were measured for each sample.

#### 2.4.4. Amino Acid Profile

The method of Awad et al. [12] was used to evaluate the content of amino acid (AA) in gelatin samples. A total of 5 mL of HCl (6 mol/L) was added to approximately 0.1 g samples and kept in a hot air oven for 24 h at 110 °C for hydrolysis. High-performance liquid chromatography (HPLC) (Waters Corporation, Milford, MA, USA) was performed following pre-column derivatization with the 6-aminoquinolyl-*N*-hydroxy succinimdyl carbamate. To the hydrolysate, 4 mL of *L*-α-amino-*N*-butyric acid (AABA), used as internal standard, was added, and the volume reached 100 mL by adding dH_2_O. The mixture was paper- and syringe-filtered. A total of 70 μL borate buffer and 20 μL of ACCQ reagent were added to 10 μL of the filtrate. Fluorescent detector (2475; Waters Corporation, Milford, MA, USA) was used for peaks detection. Data expressed were mean values, and standard deviations were lower than 2% in each case.

#### 2.4.5. Sodium Dodecyl Sulphate–Polyacrylamide Gel Electrophoresis (SDS-PAGE)

Electrophoretic analysis was performed to visualize the molecular weight distributions of the extracted gelatins using Mini-PROTEAN Tetra System purchased from Bio-Rad Laboratories, Irvine, CA, USA. The sample solution containing β-mercaptoethanol was added to loading buffer in a 1:2 (*v*/*v*) ratio and loaded onto 4% stacking gels and 7.5% resolving gels. Initially, a constant current of 15 mA/gel was used for 15 min to run the gel system followed by 25 mA/gel until the dye bromophenol blue reached at the lower edge of the gel. BLUeye ladder from GeneDireX, Taiwan, was run concurrently to determine the molecular weight of the gelatin proteins.

#### 2.4.6. Free Amino Acid Content

The procedure earlier described for amino acid content determination was followed to analyze free amino acid content of gelatins, except that the 6 N HCl hydrolysis of the samples were not performed. About 100 mg of sample was weighed and directly mixed with 4 mL of internal standards (AABA). The rest of the steps were similar.

#### 2.4.7. Gel Strength

Gel strength measurement was carried out according to slightly changed method of Fernandez-Dıaz [13] using Stable Micro Systems texture analyzer (Model TA-XT2*i*, Surrey, UK) using a load cell of 5 kN. We made the 6.67% (*m*/*v*) gel solution in 60 °C distilled water using a beaker (50 mL) (SCHOTT DURAN, Mainz, Germany), which was kept at 7 °C for 16–18 h for gel maturation. The diameter and height of the samples were 3.8 and 2.7 cm, respectively. Teflon-coated cylindrical plunger (P/0.5R) with a flat-face and 1.27 cm diameter was attached to the analyzer to determine the gel strength. While taking the measurements, we kept the plunger speed at 0.5 mm/s, and maximum force (grams) required to pierce 4 mm deep inside the sample was recorded as gel strength.

#### 2.4.8. Turbidity

Turbidity was estimated following the procedure of Cho et al. [14]. Sample solution (0.5% (*m*/*v*)) was prepared by dissolving gelatin in distilled water heated to 60 °C. Kaolin solution was taken as standard. The observances were taken at 660 nm using a Shimadzu UV Spectrophotometer (Model UV-1800, Nakagyo-ku, Kyoto, Japan).

#### 2.4.9. Viscosity

A total of 1.34 g of gelatin was weighed and added to 20 mL of distilled water to create a solution of 6.67% (*m*/*v*). The mixture was heated to 60 °C to dissolve the gelatin completely. The viscosity of the samples was measured by RheolabQC (Anton Paar, Graz, Austria) viscometer at 25 °C.

#### 2.4.10. ^1^H NMR Measurements

An NMR spectrometer (Varian INOVA) of Varian Inc., Walnut Creek, CA, USA, was used at room temperature (24 °C) at 500 MHz frequency to obtain proton nuclear magnetic resonance (^1^H NMR) of the samples. A total of 1 mg of gelatin was solubilized in 0.5 mL of deuterated water (D_2_O) (KH_2_PO_4_ buffer, pH 6) containing 0.01% dimethyl silapentane sulfonic acid (DSS) as a reference compound. The spectral processing of NMR data was performed using Chenomx NMR Suite software (Version 7.1, Chenomx Inc., Edmonton, AL, Canada). Spectral width was kept at 20 ppm and spectral acquisition time for each sample was 3.53 min consisting of 64 scans.

#### 2.4.11. FTIR Spectroscopy

An FTIR spectrometer purchased from Shimadzu, Kyoto, Japan (model IRTracer-100), was utilized to acquire FTIR spectra of gelatin samples. Attenuated total reflectance (ATR) accessory was fitted to sample compartment. Deuterated L-alanine triglycine sulphate (DLATGS) was used as detector. Internal reflection crystal made of CsI had a 45° angle of incidence to the IR beam. The spectra were taken in 4000–650 cm^−1^ (mid-IR region) keeping the resolution of 4 cm^−1^. A clean and empty cell was used to obtain background spectrum at 25 °C against which the signals were normalized. Automatic signals were collected in 16 scans.

### 2.5. Statistical Analysis

All the analyses were carried out in triplicate. Statistical Analysis System package (SAS) Version 9.4 software (SAS Institute Inc., Cary, NC, USA) was used for all statistical analysis and data were subjected to ANOVA analysis where the different levels of enzyme were fixed as the main effects. Statistical difference was considered significant at *p* < 0.05.

## 3. Results and Discussion

### 3.1. Effect of Pepsin on Gelatin Recovery

The gelatin extraction yield increased significantly (*p* < 0.05) from 17.90% (control gelatin) and 18.17% (Pe5) to 22.79% (Pe15) and 24.67% (Pe25) as the level of pepsin enzyme increased (Table 1). Augmented gelatin yield of 30% was obtained from skin of thornback ray treated with commercial pepsin (5 units/g of skin) [6]. Bigeye snapper pepsin (BSP) used at the level of 0 and 15 units/g of alkaline-treated bigeye snapper skin resulted in corresponding gelatin recovery of 22.2 and 40.3%, respectively [4]. Increasing the enzyme level from 5 to 15 U of pepsin/g of alkali-pretreated cuttlefish (*Sepia officinalis*) skin resulted in improvement in gelatin yield from 6.59 to 9.22%, respectively, and without pepsin, the gelatin recovery was only 2.68% [5]. The amount of collagen converted to gelatin was 32% with the pretreatment of bovine skin with pepsin [7]. The collagen inter- and intramolecular cross-links are resistant to heat and acid [3,15]. These bonds are primarily located on the telopeptide region of the triple helical structure of the collagen [16] and are efficiently broken down by pepsin [4]. Thus, pepsin loosens the collagen protein chain matrix and aids in the greater recovery of gelatin [4]. Similarly, Chomarat et al. [7] reported that treatment of collagen with pepsin under non-denaturing conditions had little effect on the native triple helical structure of collagen and proteolytic pepsin acts on the intra- and intermolecular cross links present at the non-collagenous terminal ends and thereby convert the non-soluble collagen into a soluble form [17].

### 3.2. pH

The pH values of the control and three gelatin samples showed non-significant (*p* > 0.05) differences (Table 1). Nevertheless, in the present study, pH was found to be low compared to earlier reported values of commercial bovine gelatins. The pH values of commercial bovine gelatins were mentioned as being 5.73 [18] and 5.48 [19]. The difference of pH for the gelatin obtained in this study and earlier reports might have been due to acidic (HCl) treatment of bovine skin and alkaline treatment given to commercial gelatins during their extraction [18]. Non-significantly (*p* > 0.05) lower pH of control gelatin (2.16) could have been due to presence of higher amount of aspartic and glutamic acid in control gelatin in comparison with the Pe5, Pe15, and Pe25 gelatins. During gelatin extraction for longer duration, deamidation might occur, resulting in conversion of asparagine and glutamine into aspartic acid and glutamic acid, respectively [11].

### 3.3. Color

Color of the gelatin is dependent on the raw material and extraction condition [18]. Color is established to be important aesthetic characteristics of gelatin depending on the intended use [6], but color does not influence the functional properties of the gelatins [9]. Lassoued et al. [6] reported L*, a*, and b* values as 42.71, −0.93, and 15.86, respectively, for food-grade halal bovine gelatin. These values are comparable to values obtained in this study. The significantly (*p* < 0.05) higher L* value for the control, Pe15, and Pe25 in comparison with Pe5 showed that the control, Pe15, and Pe25 are comparatively lighter in color than Pe5 gelatin (Table 2). a* (redness) and b* (yellowness) values were significantly (*p* < 0.05) higher for Pe15 and Pe25 in comparison to Pe5, indicating that Pe15 and Pe25 were more red and yellow compared to Pe5. The b* value (yellowness) of gelatin extracted from zebra blenny skin using crude acidic protease obtained from its viscera was found to be 13.97, and the gelatin was found to be a light-yellow color [9]. Finally, the research concluded that such color was suited for various foods since it would not impart any strong color to the final product. Similarly, in the present study, different gelatin samples could be said to possess light yellow color since b* values fell in the range between 5.65 and 12.12.

### 3.4. Amino Acid Profile

Amino acid profile of the three treatment gelatins (Pe5, Pe15, and Pe25) is presented in Table 3. Glycine, proline, and hydroxyproline were reported to be 17.96 and 20.60%, 8.81 and 21.23%, and 4.21 and 9.80% by Mulyani et al. [20] and Aykın-Dinçer et al. [18] in bovine skin gelatin, respectively, whereas in the present study, the corresponding values varied from 21.56 to 21.47%, 10.46 to 10.51%, and 14.87 to 14.76%, respectively, for the three treatment gelatins Pe5, Pe15, and Pe25. Control gelatin has an amino acid profile similar to the treatment group. Differences in the gelatin manufacturing process [21] and different acid pretreatment processes resulted in variation in the amino acid content [22]. Mulyani et al. [20] reported much less imino acid (proline + hydroxyproline) content (13.02%) in bovine skin gelatin in contrast to imino acid content (varying from 25.33 to 25.27%) in the treatment gelatins obtained from bovine skin in this study. Nevertheless, the imino acid content mentioned by Aykın-Dinçer et al. [18] in bovine skin gelatin was 31.03%. The main reason for such a large difference compared to values in the current study was low hydroxyproline content reported by Mulyani et al. [20] and high proline found by Aykın-Dinçer et al. [18]. Hydroxyproline is mainly involved in providing the stability to the triple helical structure of the renatured gel by its ability to form H-bonding through hydroxyl group [23,24]. Even though the imino acid content (particularly, hydroxyproline) was found to be higher, the corresponding high gel strength was recorded only for control (283.35 g) and Pe5 (215.49 g) gelatin. The gel strength of bovine skin gelatin, as stated by Mulyani et al. [20] and Aykın-Dinçer et al. [18], were 208.26 and 238.25 g, respectively. Viscoelastic characteristics of gelatin is not only dependent on the imino content of the gelatin [25] but also dependent on the complex interactions controlled by the molecular weight distribution [16], as well as on the length of protein chain fragments, as short chain polypeptides fail to form the junction zone in which strong network is developed [26].

### 3.5. SDS-PAGE Analysis

**Figure 1:**
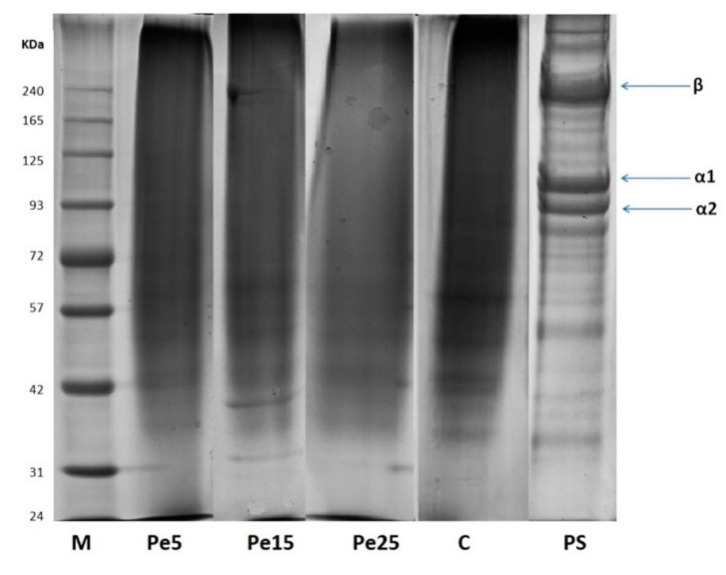
SDS-PAGE pattern of gelatin samples extracted using different levels of pepsin enzyme. Pe5, Pe15, and Pe25 denote gelatins extracted using enzyme levels of 5, 15, and 15 unit/g of skin, respectively; C refers to control gelatin extracted without pepsin; PS denotes pretreated skin sample; M denotes high molecular marker.

The protein pattern of gelatins recovered from bovine skin using pepsin enzyme pretreatment is shown in Figure 1. Molecular weight distribution of polypeptide protein chains, their structure, and subunits composition greatly affects the functional characteristics of gelatin in addition to amino acid content [27]. β-Chains (dimers of α-chain) as well as α1 and α2 chains of all the gelatin samples underwent complete degradation. Protein fragments lower than 72 kDa were detected in control gelatin. Only short chain proteins of molecular weight lower than 42 kDa were present in samples Pe5 and Pe15. Pe25 revealed only smear bands. Pretreated skin sample clearly showed presence of β- and α (α1 and α2)-chains in addition to many short peptide bands of degraded protein chains.

Polyacrylamide gel elucidated only 21 and 14 kDa protein bands for gelatin obtained from raw hide by commercial papain hydrolysis [28]. Inter- and intra-molecular bonds present in collagen are broken down by hydrolysis, resulting in the presence of low-molecular-weight polypeptide ranging from 10 to 250 kDa in gelatin [29]. Low-molecular-weight protein fragments were also observed in gelatin by Weng et al. [30]. Collagen protein chains are degraded into low molecular weight components during its conversion to gelatin [31]. Gelatin extracted from zebra blenny skin showed the presence of low-molecular-weight protein fragments attributed to over hydrolysis caused by zebra blenny crude acid protease obtained from its viscera [9]. Increasing the pepsin concentration for gelatin extraction from skin of cuttlefish led to the reduction in the intensity of α1- and α2-chains [5]. Additionally, extensive degradation of protein by pepsin was held responsible for occurrence of low-molecular-weight peptides in gelatin samples, and thus pepsin affected the protein chain length of the extracted gelatin [5]. Gelatins obtained from bigeye snapper skin using increasing level of bigeye snapper pepsin recovered from its stomach revealed complete disappearance of β, α1, and α2 components, and only smear bands were observed [4].

### 3.6. Free Amino Acid Content

**Figure 2:**
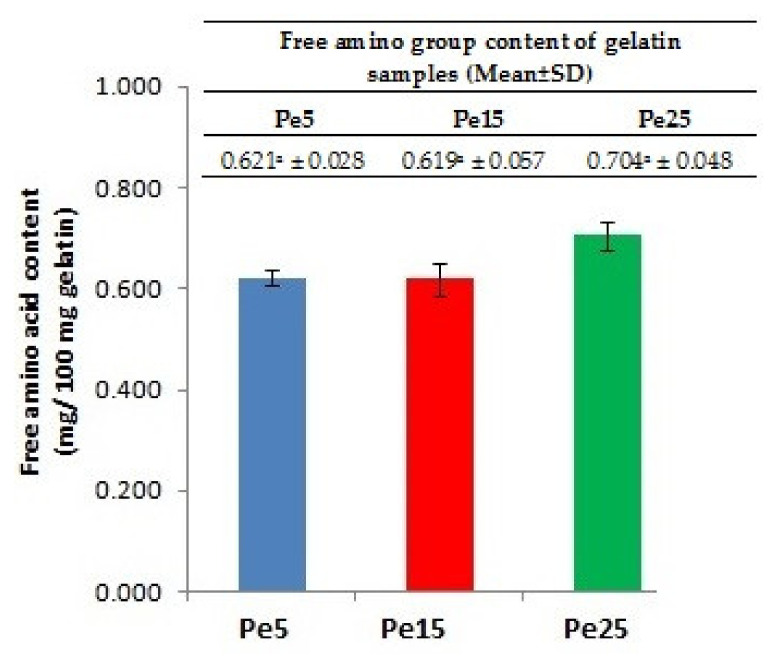
Effect of different levels of pepsin on free amino group content of gelatins extracted from the bovine skin. Pe5, Pe15, and Pe25 denote gelatins extracted using enzyme levels of 5, 15, and 15 unit/g of skin, respectively. Bars represent the standard deviation (*n* = 3).

The results for the amino group content in the different gelatin samples are shown in Figure 2. The higher free amino acid content in Pe25 compared to Pe5 and Pe15 correlated well with the SDS-PAGE image, which revealed extensive degradation of proteins molecules resulting in only smear band. The higher level of pepsin used in Pe25 extraction might have led to severe degradation of protein chains, resulting in higher amount of free amino acids in Pe25. Increasing amount of free amino group was found in the gelatin sample extracted at high temperature due to cleavage of proteins [24]. Similarly, higher free amino groups were detected in gelatin samples extracted using ultrasound and longer time duration [32].

### 3.7. Gel Strength

Gel strength is one of the most essential functional attributes of gelatin [18,23] and is greatly dependent on inherent properties such as amino acid composition, molecular weight distribution, and extraction procedure deployed to extract the gelatin [22]. The gel strengths of various gelatin samples extracted from bovine skin using pepsin along with control are presented in Table 1. The gel strength of gelatins decreased significantly (*p* < 0.05) from 283.35 g for the control to 215.49 g for Pe5 and 56.06 g for Pe25 as the pepsin concentration increased. The result findings are similar to the reported gel strength of gelatins extracted using incremental level of pepsin [5]. The researchers found that the gel strength of gelatin decreased from 197 to 120 g as the pepsin concentration increased from 5 to 15 U pepsin/g cuttlefish skin. Gelatins extracted at different pH values of 8, 7, and 6 from rawhide splits employing papain showed corresponding gel strength of 200.6, 146.5, and 75.4 g, respectively [33]. The gel strength of commercial gelatin from cow hide procured from the Brazilian market was found to be 204.05 g [34].

Higher concentration of pepsin resulted in destruction of α-chains at the telopeptide region, resulting in low-molecular-weight components ensuing to low gel strength [5]. Although imino acid content was high in gelatin recovered from squid, it exhibited gel strength less than 25 g as it contained degradation protein fragments of lower molecular weight [16]. Gelatin extracted from bigeye snapper skin had low bloom value (56 g), owing to the presence of shorter length protein chains [4]. These degraded products of α- chain fragments hamper the growth of already available nucleation sites and thus fail to anneal correctly during the overnight maturation period, resulting in low bloom value [26]. The similar SDS-PAGE pattern and imino acid content for Pe5 and Pe15 but significantly lower gel strength of Pe15 could be due to inability of the protein chain fragments to form a fine and ordered gel structure during gel renaturation [23]. Pe15 and Pe25 could be used in the fruit juice industry for clarifying juice as low bloom gelatins are found to be suitable for such purposes [6].

### 3.8. Turbidity

Significant (*p* < 0.05) increase in the turbidity of gelatin samples from control to Pe5 and Pe25 (Table 1) was well correlated with the decrease in gel strength, as also reported by Kittiphattanabawon et al. [26]. Disordered aggregation of gelatin protein chains might result in high viscosity as haphazard alignment of gelatin molecules produce the translucent-type gel associated with high turbidity [26]. As discussed earlier, random aggregations of gelatin protein fragments during gelatins also results in low gel strength.

### 3.9. Viscosity

The significantly (*p* < 0.05) higher viscosity of 12.10 and 9.17 mPa·s was exhibited by control and Pe5 gelatin sample, respectively, compared to Pe15 (8.10 mPa·s) and Pe25 (8.17 mPa·s) gelatins (Table 1). There is a difference in the report of viscosity of bovine gelatin by different researchers. Mohtar et al. [19] reported the viscosity of commercial bovine gelatin (Type A, Bloom 200, sample no. 378-1208 from International Food Agencies Limited, Auckland, New Zealand) to be 9.80 cP, whereas it was found to be 4.5 mPa for bovine gelatin samples obtained from Halalgel Sdn. Bhd., Malaysia, by Norziah et al. [35]. Rawhide gelatins extracted at different pH values of 6, 7, and 8 with the use of papain showed corresponding viscosities of 3.5, 4.5, and 5.6 Pa s^−1^ [33].

Being the second most important functional characteristics after gel strength [36], gelatin with high viscosity is considered commercially more valuable [21]. Protein chain molecular weight as well as their distribution partially determines the viscosity of gelatin [37]. The significantly (*p* < 0.05) higher viscosity exhibited by the control and Pe5 compared to Pe25 was due to presence of higher molecular protein fragments. Although the SDS-PAGE image revealed the presence of similar molecular weight protein and their distribution, the viscosity of Pe15 was significantly (*p* > 0.05) lower than Pe5. This lower viscosity could be explained on the basis that not only molecular weight of gelatin protein fragments but their polydispersity affects the viscosity to a great extent [38], denoting that although higher viscosity is exhibited by gelatin possessing a higher molecular weight component, polydispersity can have a variable effect depending on the distribution of protein chain molecular weight [39], resulting in less viscosity of Pe15 than Pe5.

### 3.10. ^1^H NMR Measurements

**Figure 3:**
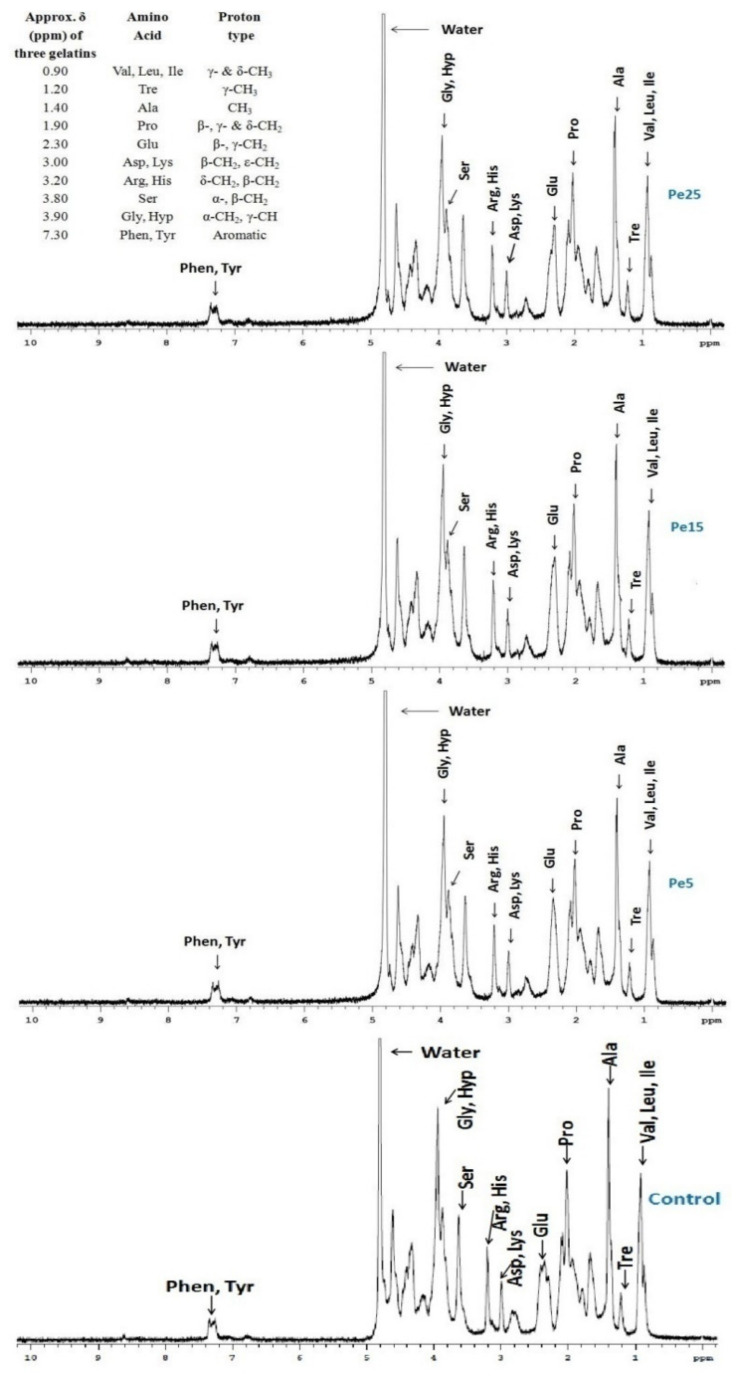
^1^H nuclear magnetic resonance (NMR) spectra of gelatins extracted using different levels of pepsin enzyme. Pe5, Pe15, and Pe25 refers to gelatin extracted using pepsin at the levels of 5, 15, and 25 unit/g of wet skin, respectively. Control gelatin was extracted without pepsin.

The proton NMR spectra for control, Pe5, Pe15, and Pe25 gelatins are represented in Figure 3. The spectral pattern exhibited the typical chemical environments undergone by various protons from the amino acid side chains when they were exposed to radio waves. Assignment of bands in the spectra were performed following the research articles published by Uriarte-Montoya et al. [40] and Voron’ko et al. [41] and correlated well with the amino acid composition discussed above.

The large band of spectra at δ = 5.0 suggested the presence of water molecules in the coiled protein chain structure of the gelatin sample. These water molecules might be encircling the side chains of amino acids and involved in hydrogen bond formation between neighboring protein chains [42]. In contrast to singlet bands obtained for acid-soluble collagen derived from mantle squid [43], the acquired spectra were similar to spectrum obtained for giant squid skin gelatin as found by Uriarte-Montoya et al. [40]. The presence of various side chain protons in the present spectra indicated towards the coiled structure of gelatin protein chains [40]. Amino acids were loosely attached to the main protein chains, owing to few cross-linking bonds and disorder of the chains, resulting in the increase in the possibility of their interaction with solvent molecules and thus altering their individual chemical surroundings at little different frequencies in the presence of radio waves.

### 3.11. FTIR Spectra

**Figure 4:**
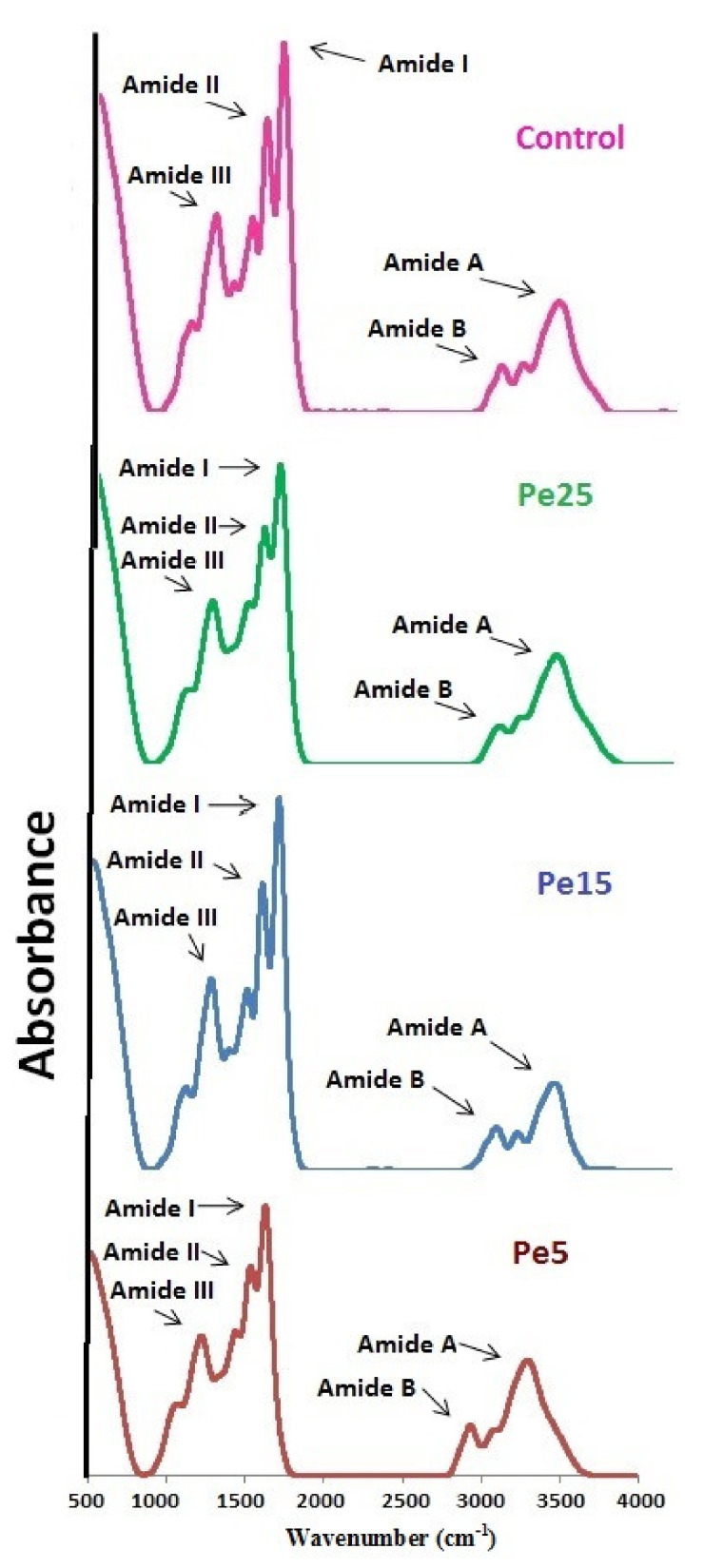
Fourier transform infrared spectra of gelatin samples extracted using different levels of enzyme pepsin. Pe5, Pe15, and Pe25 refer to gelatins extracted using enzyme levels of 5, 15, and 15 unit/g of skin, respectively. Control gelatin was extracted without pepsin.

FTIR analysis result of gelatin samples Pe5, Pe15, and Pe25 are shown in Figure 4, and corresponding spectra peak positions are represented in Table 4. With little differences in the spectra, all the gelatin samples exhibited their peaks in the amide region. Amide I bands for control, Pe5, Pe15, and Pe25 were detected at the wavenumbers 1631, 1631, 1633, and 1632 cm^−1^, respectively. Amide I band is related with C=O stretching vibration coupled to CN stretch and NH bending modes arising in the range of 1600–1700 cm^−1^ [44]. Amide I band denotes bonding of C=O group with nearby chains through hydrogen bonding [45]. Shift of amide I to higher wavenumber combined with high amplitude denotes greater loss of molecular order. Thus, there was greater loss of molecular order in Pe15 followed by Pe25. Shifting of amide I to higher wavenumber showed higher degradation of protein chains in Pe15 and Pe25 into smaller fragments [44].

Amide II band is established to be associated with in-plane bending modes of N–H of the peptide groups and stretching vibration of C–N groups [24]. Lower wavenumber along with lower amplitude of amide II indicate higher involvement of NH groups in hydrogen bond formation with neighboring chains [46]. The characteristics amide II band of control, Pe5, Pe15, and Pe25 were exhibited at the wavenumbers 1537.27, 1536.71, 1534.61, and 1535.21 cm^−1^, respectively. Although amide II peak of Pe15 was lowest among the four gelatins, its amplitude was higher than other two samples Pe5 and Pe25. Among Pe5 and Pe25, lower amide II wavenumber along with lower amplitudes of Pe25 implied that comparatively more N–H peptide groups were engaged in H-bonding.

Control, Pe5, Pe15, and Pe25 exhibited their characteristic amide III peak at wavenumbers 1234, 1226, 1226, and 1224 cm^−1^, respectively. Wagging vibrations produced by CH2 groups and stretching of C–N and N–H deformations give rise to amide III band in the spectra [10]. Lower amplitude of amide III is suggestive of loss of α-helix structure to more random coiled structure [44]. The lowest amplitude was found in gelatin Pe25, pointing toward lost α-helix structure to more random coiled state during the conversion of collagen into gelatin. The higher concentration of pepsin in Pe25 might have been responsible for such a drastic degradation of native helical structure.

Amide A spectra arises due to NH-stretching coupled with hydrogen bonding, generally falling in between wavenumber 3400–3440 cm^−1^ [47]. Shifting of amide A to lower frequencies represents participation of N–H group found in the α chain of gelatin samples in hydrogen bond formation [24]. The amide A peak spectra of control, Pe5, Pe15, and Pe25 were observed at wavenumbers 3294, 3293, 3289, and 3294 cm^−1^, respectively. The lowest wavenumber of amide A for Pe15 indicated that the N–H group of a peptide was engaged in hydrogen bonding. Lower amide A as well as higher amplitude of spectra is suggestive of degradation of gelatin resulting in the presence of higher free amino group [24]. Although, the lower wavenumber was recorded for Pe15, the amplitude of amide A was also lower than the three other samples, suggesting that the gelatin degradation was not most extensive in Pe15.

Asymmetrical stretching of =C–H and NH^3+^ causing generation of vibrations results in amide B band [23]. Amide B bands (=C–H bonds) are generally detected at approximately 2930 cm^−1^. Samples showing lower wavenumber for amide B denotes higher interaction of −NH_3_^+^ groups amid peptide chains [45]. Amide B band peaks were detected at wavenumbers 2931, 2930, 2939, and 2947 cm^−1^ for gelatins control, Pe5, Pe15, and Pe25, respectively, specifying higher involvement of –NH_3_ of control and Pe5 in the interaction between peptide chains followed by Pe15.

## 4. Conclusions

Increasing level of pepsin from 5 to 25 units per gram of wet bovine skin led to increase in gelatin recovery from 18.17 to 24.67%. However, the gel strength and viscosity decreased, and turbidity increased as the pepsin level was enhanced. SDS-PAGE image showed complete degradation of β- and α-chains and presence of lower molecular weight protein fragments in Pe5 and Pe25, whereas only smear stains were observed in Pe25. Higher level of pepsin used in Pe25 gelatin might have resulted in higher free amino acid content due to higher cleavage of protein chains. ^1^H NMR study suggested that the coiled structure of gelatin protein chains was due to the presence of various side chain protons in the three gelatins including control gelatin. Higher amplitude of amide III in Pe25 suggested conversion of α-helical configuration of collagen protein to random coiled structure during extraction.

## Figures and Tables

**Table 1:** Yields, pH, turbidity, gel strength, and viscosity of gelatins extracted from bovine skin using different levels of enzyme pepsin and control (without pepsin). Values are presented as mean ± SE from triplicate determination.

Sample	Yield (%)	pH	Gel Strength (g)	Turbidity (ppm)	Viscosity (mPa·s)
Control	17.90 ± 0.19 ^c^	2.16 ± 0.05 ^a^	283.35 ± 1.84 ^a^	12.53 ± 0.20 ^d^	12.10 ± 0.23 ^a^
Pe5	18.17 ± 0.12 ^c^	2.26 ± 0.03 ^a^	215.49 ± 2.15 ^b^	18.97 ± 0.32 ^c^	9.17 ± 0.09 ^b^
Pe15	22.79 ± 0.23 ^b^	2.23 ± 0.02 ^a^	78.58 ± 0.95 ^c^	21.07 ± 0.18 ^b^	8.10 ± 0.12 ^c^
Pe25	24.67 ± 0.31 ^a^	2.27 ± 0.05 ^a^	56.06 ± 0.99 ^d^	24.00 ± 0.46 ^a^	8.17 ± 0.03 ^c^

^a, b, c, d^ Means with different superscripts in the same column indicate significant difference at *p* < 0.05. Pe5, Pe15, and Pe25 refer to gelatin extracted using pepsin at the levels of 5, 15, and 25 units per gram of wet skin, respectively.

**Table 2:** Color of gelatins extracted from bovine skin using different levels of enzyme pepsin and control (without pepsin). Values are presented as mean ± SE from triplicate determination.

Sample	L*	a*	b*
Control	68.19 ± 0.14 ^a^	1.10 ± 0.06 ^c^	9.52 ± 0.11 ^b^
Pe5	47.57 ± 1.15 ^d^	1.19 ± 0.09 ^c^	5.65 ± 0.08 ^c^
Pe15	52.28 ± 0.26 ^c^	2.19 ± 0.07 ^a^	12.12 ± 0.10 ^a^
Pe25	58.71 ± 0.08 ^b^	1.92 ± 0.01 ^b^	10.86 ± 0.04 ^b^

^a, b, c, d^ Means with different superscripts in the same row indicate significant difference in the color coordinates at *p* < 0.05. Pe5, Pe15, and Pe25 refer to gelatin extracted using pepsin at the levels of 5, 15, and 25 units per gram of wet skin, respectively.

**Table 3:** Amino acid composition (% of gelatin sample) of gelatin samples. Pe5, Pe15, and Pe25 refer to gelatin samples extracted from bovine skin using enzyme pepsin at the levels of 5, 10, and 15 unit of pepsin per gram of wet skin, respectively. Control gelatin was extracted without pepsin.

Amino Acids	Gelatin Samples			
	Control	Pe5	Pe15	Pe25
Hydroxyproline (Hyp)	14.14 ± 1.10	14.87 ± 1.13	14.43 ± 1.13	14.76 ± 1.13
Aspartic acid (Asp)	4.06 ± 0.47	3.59 ± 0.44	3.60 ± 0.44	3.54 ± 0.36
Serine (Ser)	2.82 ± 0.58	3.25 ± 0.68	3.19 ± 0.68	3.20 ± 0.45
Glutamic acid (Glu)	7.81 ± 0.70	7.34 ± 0.58	7.28 ± 0.58	7.18 ± 0.70
Glycine (Gly)	19.87 ± 1.61	21.56 ± 1.52	21.44 ± 1.52	21.47 ± 1.61
Histidine (His)	0.82 ± 0.26	0.96 ± 0.16	0.89 ± 0.16	0.93 ± 0.23
Arginine (Arg)	6.87 ± 0.79	7.54 ± 0.79	7.24 ± 0.79	7.35 ± 0.68
Threonine (Thr)	1.63 ± 0.35	1.84 ± 0.34	1.77 ± 0.34	1.79 ± 0.45
Alanine (Ala)	6.50 ± 0.63	6.69 ± 0.70	6.73 ± 0.70	6.70 ± 0.40
Proline (Pro)	10.29 ± 0.94	10.46 ± 1.26	10.72 ± 1.26	10.51 ± 1.26
Tyrosine (Tyr)	0.62 ± 0.17	0.73 ± 0.12	0.69 ± 0.12	0.69 ± 0.14
Valine (Val)	2.11 ± 0.41	2.13 ± 0.48	2.07 ± 0.48	2.07 ± 0.50
Lysine (Lys)	3.13 ± 0.37	3.07 ± 0.74	3.02 ± 0.74	3.01 ± 0.70
Isoleucine (Ile)	1.30 ± 0.30	1.40 ± 0.31	1.34 ± 0.31	1.35 ± 0.39
Leucine (Leu)	2.66 ± 0.40	2.84 ± 0.50	2.73 ± 0.50	2.76 ± 0.38
Phenylalanine (Phe)	1.78 ± 0.32	1.91 ± 0.55	1.86 ± 0.55	1.89 ± 0.33
Imino acids (Pro + Hyp)	24.43 ± 2.03	25.33 ± 2.05	25.15 ± 0.13	25.27 ± 2.39
Total amino acids	86.41 ± 0.33	90.20 ± 2.72	88.99 ± 3.52	89.19 ± 1.45

All the data were expressed in the unit of mg/100 mg of gelatin. Measurements were performed in triplicate, and data correspond to mean ± SD.

**Table 4:** FTIR spectra peak position of gelatin samples extracted from bovine skin using pepsin enzyme at the levels of 5, 15, and 25 unit/g of wet skin, and therefore the recovered gelatins were referred to as Pe5, Pe15, and Pe25, respectively. Control gelatin was extracted without pepsin.

Region	Peak Wavenumber (cm^−1^) of Gelatins			
	Control	Pe5	Pe15	Pe25
Amide I	1631.78	1631.83	1633.70	1632.92
Amide II	1537.27	1536.71	1534.61	1535.21
Amide III	1234.44	1226.02	1226.92	1224.26
Amide A	3294.42	3293.23	3289.73	3294.53
Amide B	2931.80	2930.84	2939.95	2947.89

## Data Availability

The data presented in this study are available on request from the corresponding author. The data are not publicly available due to privacy issues.
